# Improvement of Pancreatic Steatosis and Indices of Insulin Resistance After Metabolic Surgery

**DOI:** 10.3389/fmed.2022.894465

**Published:** 2022-06-06

**Authors:** Ahmed Abdallah Salman, Mohamed Abdalla Salman, Mostafa Said, Mohammad El Sherbiny, Hesham Elkassar, Mohamed Badr Hassan, Ahmed Marwan, Mohamed Abdelkader Morad, Omar Ashoush, Safa Labib, Mohamed H. Aon, Abeer Awad, Mohamed Sayed, Ahmed E. Taha, Ahmed Moustafa, Hossam El-Din Shaaban, Amir Khater, Ahmed Elewa, Adel M. Khalaf, Ahmed A. Mostafa, Mohamed Matter, Ahmed Youssef

**Affiliations:** ^1^Internal Medicine Department, Faculty of Medicine, Cairo University, Cairo, Egypt; ^2^General Surgery Department, Faculty of Medicine, Cairo University, Cairo, Egypt; ^3^Internal Medicine Department, Faculty of Medicine, Mansoura University, Mansoura, Egypt; ^4^Department of Endemic Medicine and Hepatology, Faculty of Medicine, Cairo University, Cairo, Egypt; ^5^Tropical and Gastroenterology Department, National Hepatology and Tropical Medicine Research Institute, Cairo, Egypt; ^6^General Surgery Department, National Hepatology and Tropical Medicine Research Institute, Cairo, Egypt; ^7^Department of General Surgery, Faculty of Medicine, Al-Azhar University, Cairo, Egypt; ^8^Radiodiagnosis Department, Faculty of Medicine, Alazhar University, Cairo, Egypt

**Keywords:** metabolic surgery, pancreas, steatosis, insulin, resistance

## Abstract

**Purpose:**

Obesity is associated with fat accumulation in ectopic sites such as the pancreas, the so-called pancreatic steatosis (PS). Bariatric surgery has been shown to be associated with reducing pancreatic fat. This study investigated the effect of laparoscopic sleeve gastrectomy (LSG) on pancreatic volume and its fat content and glucose homeostasis.

**Methods:**

The study enrolled 54 patients subjected to LSG. Metabolic variables and pancreatic exocrine function were assessed immediately before surgery and 12 months after. MRI of the abdomen was performed to measure pancreatic fat content and its total volume and visceral adipose tissue (VAT).

**Results:**

Surgery resulted in a significant reduction in body weight and BMI. HbA1c, fasting insulin, C-peptide levels, HOMA-IR, and Hs-CRP levels decreased significantly. Surgery resulted in significant improvement in lipid profile except for HDL-cholesterol and liver function tests. Total VAT volume decreased significantly. Total pancreas volume decreased by a mean of 9.0 cm^3^ (95% CI: 6.6–11.3). The median change of pancreatic fat was −26.1% (range: −55.6 to 58.3%). Pancreatic lipase decreased significantly (*P* < 0.001). There was a positive correlation between the percentage of total weight loss and decrease in pancreatic fat volume (*r* = 0.295, *P* = 0.030).

**Conclusion:**

Weight loss after LSG is associated with a reduction of total VAT volume, total pancreatic volume, and pancreatic fat content. These changes are associated with improved glucose homeostasis, reduced systemic inflammation, and decreased pancreatic lipase secretion.

## Introduction

Obesity leads to adipose tissue dysfunction in which hypertrophic adipocytes secrete adipokines resulting in the recruitment of pre-adipocytes that differentiate into mature adipocytes (1). This process leads to fat accumulation in ectopic sites such as visceral depots, liver, skeletal muscles, and pancreas (2, 3). It was shown that visceral adipose tissue (VAT) accumulation is strongly associated with insulin resistance (IR), metabolic syndrome (MetS), predisposition to diabetes, local and systemic inflammation, and higher mortality rate (4, 5).

Fat accumulation in the pancreas is called pancreatic steatosis (PS), the most common benign pathologic condition of the pancreas in adults (6). It was demonstrated that nearly 2/3 of obese individuals has pancreatic fat deposition (7). Pancreatic fat content is significantly correlated with higher body mass index (BMI) and advanced age (8). Besides, ectopic pancreatic fat has been linked to β-cell dysfunction and type-2 diabetes mellitus (T2DM) (9, 10). Also, PS was shown to accelerate acute pancreatitis due to elevated levels of lipolysis and inflammation (11).

Bariatric surgery is widely recognized as the most effective treatment approach for obesity with a relatively low odds of obesity remission (12). Patients may lose nearly half of their subcutaneous and visceral fat within the first year after surgery (13). Subsequent weight loss is mainly from visceral depots, which correlates with the extent of diabetes remission (14). Some reports have shown a reduction of ectopic pancreatic fat following bariatric surgery, an effect that might promote improved glucose metabolism (14–16).

This study aimed to explore the effects of weight loss after laparoscopic sleeve gastrectomy on pancreatic volume and its fat content and glucose homeostasis.

## Subjects and Methods

The study enrolled 54 subjects with morbid obesity who underwent laparoscopic sleeve gastrectomy (LSG). The inclusion criteria were an age of 18 years or more of both sexes having a preoperative body mass index (BMI) between 35 and 50 kg/m^2^. Patients were excluded if they were diagnosed with type-1 diabetes mellitus, gave a history of acute/chronic pancreatitis, were currently treated or have been treated with incretin-based therapies for T2DM, had evidence of current abuse of drugs or alcohol, or a history of severe claustrophobia. In addition, those with pacemakers, joint replacements, coronary stents, metallic splinters, nerve stimulators, cochlear implants, and pregnant women were precluded from the analysis.

### Laboratory Analysis

Metabolic variables and parameters of pancreatic exocrine function were assessed immediately before surgery and 12 months after. The glycemic variables, lipids, and liver function tests were performed following 10-h overnight fasting. Photometric assessment of glucose, lipids, and liver enzymes were performed on a Siemens Dimension VISTA 1500 System (Siemens Healthcare Diagnostics, Eschborn, Germany). Pancreatic enzymes and high-sensitivity C-reactive protein (hs-CRP) levels were assessed as pancreatic and systemic inflammation indicators. For the quantitative measurements of insulin and C-peptide on the ADVIA Centauer XP (Siemens), a two-site immunoassay was employed according to the manufacturer's directives.

A standard 75 g OGTT was carried out to determine glucose-stimulated plasma levels of GLP-1, insulin, and C-peptide. Samples were gathered at 120 min with chilled EDTA tubes containing dipeptidyl-peptidase 4 (DPP4) inhibitor. Tubes were cooled on ice water for 15 min and were then centrifuged at 3,000 *g* and 4°C for 15 min. Plasma was separated into aliquots of 500 μl each and frozen at −80°C until analysis. GLP-1 concentrations in plasma were determined by radioimmunoassay after extraction of plasma with 70% ethanol. Carboxy-terminal GLP-1 immunoreactivity was measured using antiserum 89390, which has an absolute requirement for the intact amidated carboxy-terminus of GLP-1 7–36 amide and cross-reacts <0.01% with carboxy-terminally truncated fragments and 89% with GLP-1 9–36 amide, the primary metabolite of DPP-4-mediated degradation. The sum of the two components (total GLP-1 concentration) reflects the rate of secretion of the L cell. We measured total rather than active GLP-1 as total levels reflect secretion more efficiently than levels of active GLP-1 (17). Sensitivity was below 1 pmol/L, and intra-assay coefficient of variation below 5% (18).

### Imaging Protocols

**Figure 1:**
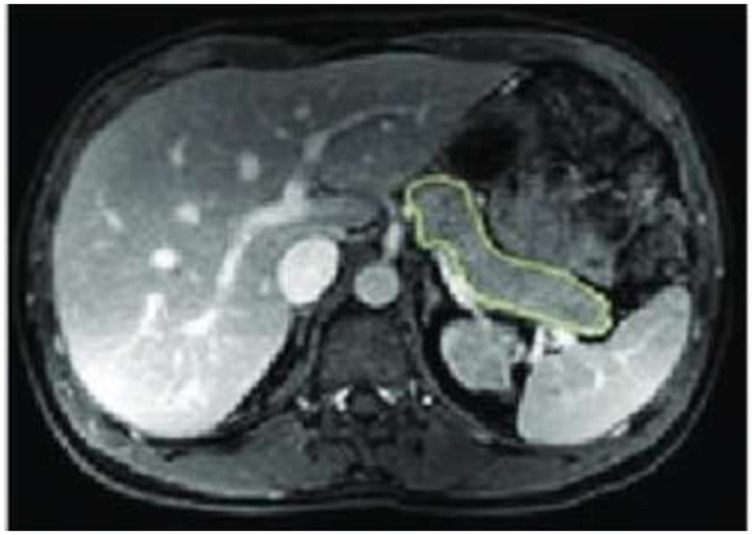
Abdominal MRI showing pancreatic size drawn by hand in the transaxial plane.

MRI of the abdomen was performed immediately before and 12 months post LSG, and no contrast media were given. Each subject was scanned once after a 10-h overnight fast in a 3 Tesla Ingenia scanner workstation (MR Systems Ingenia 3.0T, Philips Healthcare, Germany) in the supine position that first involved a set of localizer images. We employed a “Single Shot Fast Spin Echo,” which is a 3-plane localizer. Then, a respiratory-triggered T2–weighted 3D transverse fast spin-echo (TSE) sequence combined with a 2-point Dixon sequence was done. The pancreas was specified on each slice based on typical anatomical landmarks (19). Organ size was outlined by hand in each transaxial plane using OsiriX (OsiriX v.4.1, 32-bit, Pixmeo SARL), as shown in Figure 1.

For the measurement of pancreatic fat, 1–2 regions of interest (ROIs) of 0.3–0.4 cm^2^ in area were put in the head, body, and tail of the pancreas. The mean of the three selected ROIs of the pancreas was calculated to estimate the average fat fraction of the entire pancreas. Mean pixel signal intensity (SI) levels for each ROI were recorded. The fat fraction was calculated from the mean pixel SI data through the formula: Fat fraction FF (%) = [(SI in-phase – SI opposed-phase)/(2 × SI in-phase)] × 100 according to Rampton et al. (20). Pancreatic tissue fraction TF (%) was calculated as: 100% – FF (%).VAT areas of single axial slices (AVAT-1) centered at specific anatomic landmarks according to BMI and gender were calculated to predict total VAT volume (VVAT-T) as previously described (21, 22). A single experienced radiologist performed an estimation of pancreas and VAT volume.

### Statistical Methods

Statistical analysis was done using IBM© SPSS© Statistics version 26 (IBM© Corp., Armonk, NY, USA). Numerical data were expressed as mean and standard deviation or median and range as appropriate. Qualitative data were expressed as frequency and percentage. Chi-square test (Fisher's exact test) was used to examine the relation between qualitative variables. For quantitative data, comparison of repeated measures was made using paired *t*-test. Pearson product-moment was used to estimate the correlation between numerical variables. A *P*-value < 0.05 was considered significant.

## Results

All patients were available in the last follow-up visit after 12 months. The mean age of the studied group was 44.3 ± 7.0 years. They were 29 males and 25 females. Twelve patients (22.2%) had diabetes. Of them, eight were males. Mean duration of DM was 5 + 2.1 years. Of the diabetic patients, five were on insulin therapy, four on dual insulin and oral anti-diabetic drugs and three were on oral drugs only. Surgery resulted in a significant reduction in body weight and BMI (Table 1). The percentage of total weight loss (%TWL) after 12 months was 20.3 ± 4.2%.

**Table 1:** Change of weight and body mass index after surgery.

	**Before surgery (*n* = 54)**	**After surgery (*n* = 54)**	***P*-Value**
Weight (kg)	118.1 ± 10.8	94.0 ± 9.6	<0.001
Body mass index (kg/m^2^)	41.6 ± 2.6	33.3 ± 2.3	<0.001

HbA1c decreased by a median of 0.3% (range: −3.2 to 0.6%). Fasting insulin and C-peptide levels were significantly reduced after surgery (*P* < 0.001). Also, HOMA-IR decreased from 5.4 ± 0.8 to 2.8 ± 0.5 (*P* < 0.001). Seven patients showed complete remission of DM with stoppage of their medications and five showed partial remission. None of the partial remission group needed insulin therapy. Surgery also resulted in significant improvement in lipid profile except for HDL-cholesterol and liver function tests. Hs-CRP level as a parameter of chronic systemic inflammation decreased after surgery (*P* < 0.001). GLP-1 increased significantly after surgery.

### Changes in Visceral Adipose Tissue and Pancreatic Volumes

Total VAT volume significantly decreased by a mean of 2,175 cm^3^ (95% CI: 1,832–2,518). The total pancreas volume decreased by a mean of 9.0 cm^3^ (95% CI: 6.6–11.3). The median change of pancreatic fat was −26.1% (range: −55.6 to 58.3%), while that of pancreatic parenchyma was −10% (range: −27.0 to 6.9%). The baseline pancreatic fat content was 26.8 ± 7.2%, it decreased to 20.0 ± 7.3% after surgery (Table 2). The mean change was 6.8% (95% CI: 5.1–8.5).

**Table 2:** Metabolic data and laboratory parameters before and 12 months after RYGB surgery.

	**Before surgery (*n* = 54)**	**After surgery (*n* = 54)**	***P*-Value**
Fasting blood glucose (mg/ml)	103.3 ± 12.6	95.5 ± 11.8	<0.001
HbA1c (%)	5.6 ± 0.8	5.1 ± 0.2	<0.001
Fasting Insulin (mU/L)	21.2 ± 2.5	11.7 ± 1.7	<0.001
HOMA-IR Index	5.4 ± 0.8	2.8 ± 0.5	<0.001
Fasting GLP-1 (pmol/L)	20.9 ± 5.4	22.9 ± 6.7	<0.001
Fasting C-peptide (μg/L)	2.9 ± 0.5	2.4 ± 0.6	<0.001
Total VAT volume (cm^3^)	6,814 ± 1,430	4,639 ± 1,278	<0.001
Total pancreatic volume (cm^3^)	82.0 ± 9.5	73.0 ± 10.0	<0.001
Pancreatic fat volume (cm^3^)	19.3 ± 4.4	14.4 ± 4.5	<0.001
Pancreatic fat fraction (%)	26.8 ± 7.2	20.0 ± 7.3	<0.001
Pancreatic parenchymal volume (cm^3^)	66.3 ± 6.1	59.6 ± 5.6	<0.001
Fasting amylase (U/L)	20.8 ± 3.0	21.2 ± 3.6	0.472
Fasting lipase (U/L)	140.9 ± 14.9	131.4 ± 19.1	<0.001
hs-CRP (mg/dl)	0.6 ± 0.2	0.5 ± 0.1	<0.001
Total cholesterol (mg/dl)	205.6 ± 47.0	173.4 ± 32.7	<0.001
LDL-cholesterol (mg/dl)	114.4 ± 36.8	91.9 ± 23.6	<0.001
HDL-cholesterol (mg/dl)	51.1 ± 7.8	52.4 ± 7.4	0.073
Triglycerides (mg/dl)	184.2 ± 37.6	165.6 ± 27.8	<0.001
Alanine aminotransferase (U/L)	36.3 ± 7.8	26.3 ± 5.9	<0.001
Aspartate aminotransferase (U/L)	30.5 ± 6.4	23.9 ± 4.6	<0.001
Gamma-glutamyl transpeptidase (U/L)	38.8 ± 8.3	28.2 ± 5.0	<0.001
Alkaline phosphatase (U/L)	60.3 ± 9.0	60.3 ± 7.4	0.895

*HOMA-IR, homeostatic model assessment for insulin resistance; GLP-1, glucagon-like peptide-1; hs-CRP, high-sensitivity C-reactive protein; LDL, low-density lipoprotein; HDL, high-density lipoprotein; VAT, visceral adipose tissue*.

### Changes in Pancreatic Enzymes

After surgery, there was a trivial non-significant change of pancreatic amylase (*P* = 0.472). Meanwhile, pancreatic lipase decreased significantly (*P* < 0.001).

### Correlation Study

There was a positive correlation between %TWL and the decrease in pancreatic fat volume (*r* = 0.295, *P* = 0.030). Conversely, %TWL was negatively correlated with the change in pancreatic parenchymal volume (*r* = −0.278, *P* = 0.042). However, %TWL was not correlated with change of insulin, C-peptide or HOMA-IR (Table 3). C-peptide change was negatively correlated with pancreatic fat volume reduction and positively correlated with pancreatic parenchymal volume reduction. We did not find a correlation between the decrease in pancreatic fat volume and HOMA-IR drop after surgery (Table 3).

**Table 3:** Correlation between weight loss and changes of VAT and pancreatic volume and insulin resistance.

		**%TWL**	**Total VAT**	**Pancreatic fat**	**Pancreatic parenchyma**	**Insulin**	**HOMA-IR**
Total VAT	*r*	−0.173					
	*P*	0.211					
Pancreatic fat	*r*	0.295	−0.075				
	*P*	0.030	0.590				
Pancreatic parenchyma	*r*	−0.278	0.047	−0.229			
	*P*	0.042	0.737	0.095			
Insulin	*r*	0.018	0.209	−0.130	−0.19		
	*P*	0.899	0.129	0.348	0.168		
HOMA-IR	*r*	0.060	0.216	−0.205	−0.040	0.708	
	*P*	0.667	0.117	0.137	0.775	<0.001	
C-peptide	*r*	−0.253	0.234	−0.310	0.348	−0.119	−0.116
	*P*	0.065	0.089	0.023	0.010	0.392	0.404

*%TWL, the percentage of total weight loss*.

## Discussion

This study demonstrated that weight loss after LSG was associated with a significant reduction of total pancreatic volume and pancreatic fat content with a concomitant decrease of total VAT volume. These changes were associated with significant improvement of glucose homeostasis, systemic inflammation reduction, and pancreatic lipase decline. %TWL after surgery was positively correlated with pancreatic fat volume reduction and negatively correlated with pancreatic parenchymal volume reduction. Pancreatic fat volume reduction was negatively correlated with C-peptide change but not with HOMA-IR decline after surgery.

In obese subjects, fat infiltration is commonly found in the exocrine pancreas and interlobular pancreatic spaces (23). When associated with obesity, pancreatic steatosis is called non-alcoholic fatty pancreas disease (NAFPD) (24). CT image analysis demonstrates a 10%−15% increase of parenchymal pancreas mass and about 70% increase of pancreatic fat mass in the presence of obesity (25, 26).

It is logical to expect a reduction of pancreatic volume after bariatric surgery in a similar analogy to its increase with obesity. The current study found a significant decrease in pancreatic volume that was more marked in fat content compared to the parenchyma. Pancreatic fat decreased by 26.1% (range: −55.6 to 58.3%), while parenchyma decreased by 10% (range: −27.0 to 6.9%). Few studies focused on the change in pancreatic volume after bariatric surgery. Honka et al. reported a significant decrease in pancreatic fat volume and fatty acid uptake 6 months after surgery. This change was associated with favorable glucose homeostasis and improved β-cell function. The authors did not observe a significant change in pancreatic parenchyma (27). Similarly, Umemura et al. reported a significant reduction of pancreatic volume (PV) 6 months after sleeve gastrectomy. The decreased PV correlated with decreased fasting blood sugar and insulin (28). Lautenbach et al. (29) found PV reduction reversal of pancreatic steatosis to normal levels in 11 patients 6 months after RYGB, which was associated with improvement of glucose homeostasis. However, the three studies involved small sample sizes besides evaluating pancreatic changes after only 6 months. To the best of our knowledge, the current study included the largest series to demonstrate the effect of bariatric surgery on pancreatic fat after a longer follow-up period of 1 year.

We demonstrated significant improvement of glucose homeostasis concomitantly with partial resolution of pancreatic steatosis. Fasting blood glucose, insulin, and C-peptide levels were reduced significantly after surgery. Also, HOMA-IR and HA1c decreased. Many studies found that increased pancreatic fat can lead to β-cell dysfunction and insulin resistance (30–32). However, other studies reported conflicting results about the association between pancreatic fat content and beta-cell function (33, 34). It has been shown that fatty pancreas is an independent risk factor for developing DM (35). The fat fraction in the pancreatic tail was shown to significantly predict individuals at risk for the T2DM group with a sensitivity of 45.5% and specificity of 81.3% (36).

Many findings indicate a negative impact of lipotoxicity due to pancreas fat on beta-cell function. A rodent study showed that fat accumulation in pancreatic islets was associated with beta-cell dysfunction and apoptosis (37). The excess fatty acid in the pancreatic islets worsens beta-cell function and insulin secretion (38). Also, leptin, TNF-α, and adipocytokines secreted from pancreas adipocytes may induce beta-cell damage (39).

However, findings about the effects of pancreatic fat on glucose metabolism are inconsistent [74]. Histological studies did not detect significant differences between diabetic and non-diabetic subjects in pancreatic fat area (40, 41). Other reports showed that increased pancreatic fat enhances the development of glucose intolerance (12, 33). A negative association between pancreatic fat and insulin secretion has been found in subjects with a high genetic risk for diabetes (33). Also, high pancreatic fat was associated with a higher risk of T2DM in non-obese subjects (42). Other studies did not find a relationship between pancreatic fat content and beta-cell function (41, 43, 44). These inconsistencies may be due to the heterogeneous distribution of pancreatic fat. Also, most pancreatic lipids are stored in adipocytes existing in the exocrine pancreas and, to a lesser extent, in the endocrine pancreas (45, 46).

In the current study, the initial pancreatic fat content was 26.8 ± 7.2%. Surgery resulted in a mean reduction of fat content by 6.8% (95% CI: 5.1–8.5%). Previous studies reported variable pancreatic fat content in obese and normal-weight individuals. Based on pooled data from various MR methods, a meta-analysis estimated a normal mean pancreatic fat of 4.5 ± 0.9% (16). However, there is no accurate consensus on the upper limit of normal pancreatic fat content. This meta-analysis suggested a cutoff of 6.2% to differentiate normal from high pancreatic fat content (16). In a large cohort of 3,000 normal-weight adults, Wong et al. (9) reported a fat content ranging from 1.8 to 10.4%. In another study, the pancreatic fat fraction was 4.9% in normal subjects compared to 6.2 and 8.6% in those with prediabetes and diabetes, respectively (47). In a study of 267 patients, ectopic pancreatic fat was evaluated by MRI. The average fat content in the pancreas was 5.98% in patients with normal BMI, 9.36% in overweight people, and 11.69% in obese patients (48). In a recent study, the fat fraction in obese individuals was 10.3 ± 5.1% (49). An old autopsy study revealed an average of 17.1% of pancreatic fat content in obese patients compared to 9.3% fat accumulation in non-obese patients (50).

Currently, there is a number of diagnostic methods for quantifying pancreatic fat with various feasibility and reproducibility. These include ultrasonography (51), endoscopic ultrasonography (52), MRI (32), CT (51), and proton magnetic resonance spectroscopy (^1^H-MRS) (53). The most developed method is multi-echo Dixon MRI (54). MRI is considered the most accurate approach for fat quantification (55). This is contributed to the dependence of MR signal on fat content. Besides, MRI avoids the observer variability of ultrasonography and the ionizing radiation of CT (56).

The present study is not without limitations. The presence of steatopancreatitis should be better evaluated in further studies, especially regarding the inflammatory cascade. Liver volume and correlation with pancreatic fat may be needed to be assessed as well and correlated to hepatic enzymes. Also, further studies may take into account more detailed characteristics of diabetic patients such as classifications by ABCD score or IMS score and changes of beta cell function (insulinogenic index, Matsuda index, disposition index).

Despite the existence of some limitations, the current work adds a momentum to the literature regrading this novel topic. Besides, among the strengths of this work is the reasonable number of cases with a quite acceptable follow-up period. Furthermore, this work may point to potential mechanisms that may be underlying the beneficial impact of metabolic procedures on the pancreas and may provide new horizons in terms of this aspect.

We can conclude that there is an association of weight loss after LSG with a reduction of total VAT volume, total pancreatic volume, and pancreatic fat content. Weight reduction is positively correlated with the decrease of pancreatic fat content, while it is negatively correlated with pancreatic parenchymal volume reduction. The changes in pancreatic fat content are associated with improved glucose homeostasis, reduced systemic inflammation, and decreased pancreatic lipase secretion.

## Data Availability Statement

The raw data supporting the conclusions of this article will be made available by the authors, without undue reservation.

## Ethics Statement

The studies involving human participants were reviewed and approved by Cairo University Hospitals. The patients/participants provided their written informed consent to participate in this study.

## Author Contributions

All authors listed have made a substantial, direct, and intellectual contribution to the work and approved it for publication.

## Conflict of Interest

The authors declare that the research was conducted in the absence of any commercial or financial relationships that could be construed as a potential conflict of interest.

## Publisher's Note

All claims expressed in this article are solely those of the authors and do not necessarily represent those of their affiliated organizations, or those of the publisher, the editors and the reviewers. Any product that may be evaluated in this article, or claim that may be made by its manufacturer, is not guaranteed or endorsed by the publisher.
